# Supported Internet-Delivered Cognitive Behavior Treatment for Adults with Severe Depressive Symptoms: A Secondary Analysis

**DOI:** 10.2196/10204

**Published:** 2018-10-02

**Authors:** Derek Richards, Daniel Duffy, John Burke, Melissa Anderson, Sarah Connell, Ladislav Timulak

**Affiliations:** 1 Clinical Research & Innovation SilverCloud Health Dublin Ireland; 2 E-Mental Health Group School of Psychology Trinity College Dublin Dublin Ireland

**Keywords:** severe depression, internet-delivered interventions, iCBT

## Abstract

**Background:**

Depression is a highly prevalent mental health issue that exacts significant economic, societal, personal, and interpersonal costs. Innovative internet-delivered interventions have been designed to increase accessibility to and cost-effectiveness of treatments. These treatments have mainly targeted mild to moderate levels of depression. The increased risk associated with severe depression, particularly of suicidal ideation often results in this population being excluded from research studies. As a result, the effectiveness of internet-delivered cognitive behavioral therapy (iCBT) in more severely depressed cohorts is less researched.

**Objective:**

The aim of this study is to examine the effect of iCBT on symptoms of severe depression, comorbid symptoms of anxiety, and levels of work and social functioning.

**Methods:**

Retrospective consent was provided by participants with elevated scores (>28 severe depression symptoms) on the Beck Depression Inventory (BDI-II) who accessed an iCBT intervention (*Space from Depression*) with support for up to 8 weeks. Data were collected at baseline, posttreatment, and 3-month follow-up on the primary outcome (BDI-II), and secondary outcomes (the Generalized Anxiety Disorder-7 and the Work and Social Adjustment Scale).

**Results:**

A significant change was observed on all measures between pre- and postmeasurement and maintained at 3-month follow-up. Clinical improvement was observed for participants on the BDI-II from pre- to postmeasurement, and suicidal ideation also reduced from pre- to postmeasurement.

**Conclusions:**

Users of *Space from Depression* with symptoms of severe depression were found to have decreased symptoms of depression and anxiety and increased levels of work and social functioning. The intervention also demonstrated its potential to decrease suicidal ideation. Further investigation is required to determine why some individuals improve, and others do not. iCBT may have the potential to be used as an adjunct treatment for severe depression symptoms, but participants may require further treatment if they receive iCBT as a standalone intervention. Although promising, further research incorporating control groups is needed to support the utility of *Space from Depression* for use in or as an adjunct to treatment for severe depression.

## Introduction

### Background

Depression is a serious public health concern and is predicted to be the leading cause of disability in the world by 2030 [1]. Depression exacts significant economic, societal, personal, and interpersonal costs [2]. Consequently, numerous interventions, both psychological [3] and pharmacological [4], have been implemented to reduce its prevalence.

### Treatment for Depression

Evidence-based treatments for depression can comprise of pharmacological and psychological interventions [5]. Historically, pharmacological interventions have been given precedence, but psychological interventions have demonstrated their efficacy [6]. Research also suggests that two-thirds of clients with depression hold a preference for psychological interventions over pharmacological solutions [7]. However, accessing evidence-based psychological interventions can be difficult because of the many barriers [8,9], including not having access to treatment due to a lack of health service resources [8]. These barriers can be more pronounced for severe depression [10], and most individuals with severe levels of depression do not receive adequate treatment [11].

A typical diagnosis of depression as per the *International Classification of Diseases, Tenth Revision* involves an individual having at least one of 3 key symptoms of depression (depressed mood, loss of interest in daily activities, reduction in energy) for 2 weeks. Depression severity can be mediated by the presence of several other symptoms, including low self-confidence, disturbed sleep, poor concentration or suicidal thoughts [12]. The differentiation between severe depression and a mild/moderate episode is that symptoms are more pronounced and numerous, while also impairing functioning to a greater extent [13].

### Internet-Delivered Interventions

Innovative attempts to overcome these barriers have led to the development of internet-delivered psychological interventions, primarily based on cognitive behavioral principles for the treatment of depression and other mental health disorders [14]. Internet-delivered interventions can overcome some of the barriers associated with traditional treatment methods [15]. Meta-analyses have demonstrated positive results with internet-delivered cognitive behavioral therapy (iCBT) for depression [16,17], with The National Institute for Health and Care Excellence (NICE) guidelines recommending iCBT as a treatment option in the management of mild to moderate depression [13]. A legacy issue within the field of internet interventions has been that most studies have focused on participants who present within this mild to moderate symptom range, meaning the evidence base for the use of internet-delivered interventions with severe presentations is less established [18].

Nevertheless, this is changing, and some studies have demonstrated the effectiveness of iCBT in reducing symptoms of severe depression [14,19-22], with some illustrating maintenance of improvements at 6-month follow-up [19]. However, the complexities associated with delivering a successful internet-delivered intervention for this population are naturally more challenging, with some studies suggesting that severely depressed individuals participate less in iCBT [23] and sometimes demonstrate less improvement than the mild to moderately depressed group [24]. Despite this, iCBT programs tailored to each individual have been found to produce similar effects for those with severe depression, as those with mild to moderate levels [25]. Such findings have also been demonstrated in a younger cohort [23]. Progressing implementation of internet-delivered interventions with severely depressed cohorts has been much slower than in mild to moderately depressed groups, particularly because of the increased risk of suicide associated with this population [26]. Research evaluating internet-delivered interventions often exclude suicidal ideation as an ethical consideration. As such, their effects on reducing suicidal ideation and ultimately the risk of suicide are not well documented [27,28]. However, it stands that the literature in this field is slowly growing and has illustrated the benefit of iCBT for individuals with greater severity of depressive symptoms.

Where some health systems have been efficient in their adoption of iCBT and other internet-delivered interventions (the improving access to psychological therapies program in the United Kingdom), the use of iCBT in natural settings is not well documented, where iCBT may be classified as an inappropriate treatment option for severe cases. For example, the NICE guidelines for depression [13] identify iCBT and computerized therapies as appropriate treatments for mild-moderate depression, but not necessarily for more severe presentations. Despite this caution, a meta-analysis by Bower et al [14] regarding the impact of initial severity of depression on the effectiveness of low-intensity psychological interventions (both written/physical and online), concluded that those with severe presentations of depression benefit equally to those in lower severity categories. Furthermore, the ongoing monitoring of symptoms, that is facilitated seamlessly through technology, ensures accurate care delivery and informs clinical decisions on the care being delivered. These statements are further supported by the work of Watts et al [29], whose analysis of patients prescribed iCBT for depression with comorbid suicidal ideation illustrated significant decreases from pre- to posttreatment. Thus, despite the limited evidence so far, it seems appropriate, providing the intervention is safely delivered, to explore the potential of iCBT as a treatment for more severe depressive symptomatology.

iCBT can be of benefit to services as a frontline treatment for depression. As a low-intensity intervention, it consumes less clinical resource in its administration. Within a stepped care model, an individual can receive and benefit from an iCBT intervention while they wait for higher intensity treatment resources to become available. However, technology has since progressed, allowing for the delivery of treatments on more robust systems. One such intervention is SilverCloud, but the current utility of this low-intensity treatment for those with severe depression is unknown.

This study aims to explore the effect of the SilverCloud iCBT intervention on those with severe depression. It analyzes a cohort that did not meet the criteria for inclusion in a randomized controlled trial (RCT) investigating the impact of iCBT on mild to moderate depression. The authors hypothesized the following:

- The intervention could impact positively on self-reported symptoms of depression, anxiety and work, and social functioning within a cohort with severe depression symptoms.
- The results of this study offer a unique opportunity to assess the effects on suicidal ideation in the cohort, and the authors hypothesized that suicidal ideation would be lower post intervention.
- Clinically meaningful change in depressive symptoms will be observed for the group.

## Methods

### Design

Participants in this secondary analysis consisted of a subsample from a larger RCT [27,28]. The larger RCT recruited participants who were within the mild to moderate symptom severity as determined by a score of between 14 and 28 on the Beck Depression Inventory (BDI-II). Those scoring above these criteria on the BDI-II (ie, within the “severely” depressed range) were excluded from the main RCT study but were offered access to treatment with support and invited to complete posttreatment and follow-up questionnaires. Individuals scoring in this range also received a referral to the general practitioner as per risk protocols. No case-control group was recruited for this cohort as it extended beyond the scope of the main research study.

### Participants and Recruitment

Table 1 describes the sociodemographic characteristics of the sample. Participants of the present study scored within the “severe” range for depression (>28 on the BDI-II) [30]. As part of the original RCT protocol, participants were recruited via outreach work and information on the website of Aware, a national depression charity in Ireland. Two-hundred and eleven (N=211) participants were excluded from the main RCT due to symptom severity. An ethical amendment was submitted and approved at a later date to contact these individuals via email to request permission for their data to be analyzed as part of the current secondary analysis. Of the 211, 67 (32%) participants replied and consented for their data to be used in the secondary analysis of outcomes. In total, 67 participants provided data at baseline on all measures, with responses on measures from 33 (49%) participants on the BDI-II and Work and Social Adjustment Scale (WSAS), 34 (51%) on the Generalized Anxiety Disorder-7 item inventory (GAD-7) at post treatment, and from 22 (33%) on all measures at 3-month follow-up.

### Measures

Participants were assessed at baseline and posttreatment (after 8 weeks of service provision) and again at 3-month follow-up. At baseline, the BDI-II, Sociodemographic and History Questionnaire, GAD-7, and the WSAS were completed for screening purposes. After that, the BDI-II, GAD-7, and WSAS were completed at the end of treatment, week 8, and at 3-month follow-up. The 21-item BDI-II [30] is a widely used questionnaire measuring symptoms and severity of depression based on the criteria for depressive disorder diagnosis as outlined in The American Psychiatric Association *Diagnostic and Statistical Manual of Mental Disorders,* Fourth Edition (DSM-IV) [31].

**Table 1:** Participant demographics (N=67).

Characteristic		Value
Age (years), mean (SD); range		36.3 (10.4); 18-58
**Gender, n (%)**		
	Male	8 (12)
	Female	59 (88)
**Education level**		
	High school	13 (19)
	Undergraduate	30 (45)
	Postgraduate	7 (10)
	Other	14 (21)
	None	3 (4)
**Employment status**		
	Part-time/student	14 (21)
	Full-time	25 (38)
	Unemployed	15 (22)
	Retired	1 (2)
	Disabled	2 (3)
	Stay-at-home parent	10 (15)

The scale designates levels of severity: minimal (0-13), mild (14-19), moderate (20-28), and severe (29-63). The BDI-II has been found to have excellent internal consistency and test-retest reliability with a diverse range of samples [30,32,33]. It has also demonstrated good convergent validity with other measures of depression among clinical and nonclinical adult samples [34]. Reliability analysis of this measure for the current sample indicated appropriate levels of internal consistency (alpha=.71).

The GAD-7 [35] comprises 7 items measuring symptoms and severity of anxiety based on the DSM-IV diagnostic criteria. The GAD-7 has good internal consistency (alpha=0.89) and good convergent validity with other anxiety scales [36]. The GAD-7 is increasingly used in large-scale studies as a generic measure of changes in anxiety symptomatology, using a cutoff score of 8 [37,38]. Reliability analysis of this measure for the current sample indicated appropriate levels of internal consistency (alpha=.84).

The Work and Social Adjustment Scale (WSAS) [39] is a simple, reliable, and valid measure of impaired daily functioning across five dimensions: work, social life, home life, private life, and close relationships. Reliability analysis of this measure for the current sample indicated appropriate levels of internal consistency (alpha=.72).

### The Intervention

The SilverCloud *Space from Depression* program is a 7-module, internet-delivered cognitive behavioral program, with an option to unlock further content as the user progresses. Program content is delivered on a web 2.0 platform and features several forms of rich media (videos, animations, and audio) to facilitate the delivery of the intervention. Treatment content consists of cognitive and behavioral strategies common to CBT protocols: behavioral activation, cognitive restructuring, and activity scheduling [28]. For the current trial, the program was delivered in a supported format and participants received brief asynchronous feedback based on their progress each week for a period of up to 8 weeks. A detailed description of the modules is provided in Table 2.

Trained volunteers from a national depression charity in Ireland provided support for the intervention. The role of the supporter in this trial was to provide motivational support and encouragement to their assigned patient. The cohort undertook several face-to-face training sessions hosted by the charity before commencing in their role, with the content of the training designed to educate on CBT, the program, the role of the supporter, and how best to respond to patients in distress. An assistant psychologist was employed by the depression charity to assist the supporters, as well as to monitor all correspondence between supporters and their patients. This individual reported directly to the Education and Online Services coordinator, who regularly consulted with the clinical director of the charity. Further risk protocols were incorporated at review points, where a client was escalated along service protocols if they shared information with their supporter that indicated a risk to themselves or others. Where patients indicated a response greater than zero on item 9 of the BDI-II, the supporter was sent an email alert, and clients were presented with “get help now” links and local crisis numbers. This protocol was standard for all patients of the charity, and not just those in the severe group. The supporter would then contact the patient at their earliest convenience, and they would discuss the options available to them.

**Table 2:** Contents of *Space from Depression* and brief descriptions of modules.

Module name	Brief description
Getting started	Outlines basic premise of CBT, provides information about depression, and introduces some of the key ideas of *Space from Depression* Users are encouraged to begin to chart their current difficulties with depression
Tune In I: getting to grips with mood	Focus is on mood monitoring and emotional literacy Users can explore different aspects of emotions, physical reactions, action and inaction, and how they are related
Tune In II: spotting thoughts	Module focuses on noting and tracking thoughts Users can explore the connection between their cognitions and their mood, and record them graphically
Change It I: boosting behavior	Module focuses on behavioral change as a way to improve mood Ideas about behavioral activation are included, and users can plan and record activities, and chart their relationship with their mood
Change It II: challenge your thoughts	Module supports users to challenge distorted or overly negative thinking patterns, with thought records, as well as helpful coping thoughts
Change It III: core beliefs	Module outlines the role that deeply held core beliefs could play in mood and depression A range of interactive activities available to identify, challenge, and balance any unhelpful core beliefs
Bringing it all together	Module encourages bringing together all skills and ideas they have gathered so far, note their warning signs, and plan for staying well

### Ethical Approval

Original ethics approval was received for the study on November 25, 2013. Posthoc ethical approval was granted on November 16, 2015, to contact those scoring in the severe ranges of the BDI-II. The subsample was provided with information on the secondary analysis and participants were requested to provide informed consent should they wish to participate.

### Data Analysis

Baseline comparisons across the variables of age, gender, education level, and employment status were conducted, along with baseline comparisons across the measures of BDI-II, GAD-7, and WSAS between data gathered from all clients with “severe” depression (N=211) as per the BDI-II, and those who consented for their data to be used in the follow-up analysis (n=67).

Multivariate imputation by chained equations was applied to impute missing question scores using the R MICE v2.0 package [40]. Variables were then imputed with 100 imputations for 30 iterations using predictive mean matching. Following imputation, linear mixed models were fitted for each of the three measures (BDI-II, GAD-7, and WSAS) using the R package lme4 v1.1-17 [41], pooling results over the 100 individual datasets. This method was chosen due to its robustness in the handling of missing data, as this study recorded 49% (33/67) missing data at posttreatment and 68% (46/67) missing data at 3-month follow-up. F tests for fixed effects were carried out with the ANOVA (analysis of variance) function of the R package ImerTest [42], using Satterthwaite’s method for denominator degrees-of-freedom approximation. Effect sizes (Cohen *d*), averaged over all imputed datasets, were calculated using the mean change and standard deviation of change scores which is a recommended method for repeated measures designs [43-45]. Clinically significant change and deterioration were calculated based on a movement of 9 or more points on the BDI-II from pre- to posttreatment measurement [46]. Reliable recovery was calculated based on a movement of 9 more points, as well as having a posttreatment score of less than 10 on the BDI-II [30]. As per this methodology, 4 categories were established: reliable improvement, reliable deterioration, reliable recovery, and no change.

## Results

### Baseline Characteristics

No significant differences were observed between the BDI-II, GAD-7, and WSAS using *t* tests between the overall cohort with severe depression (N=211) and those who consented (n=67) to having their posttreatment and follow-up data included in the analysis. Across demographic variables, no significant differences in gender, employment status or age were observed at baseline. However, a significant difference was observed on the level of education between the 2 groups (*χ*^2^_4_=10.0, *P*=.04), with the consenting participants having a higher level of education overall.

### Treatment Response

Participants engagement with the treatment was positive. To begin with, the participants completed a mean of 17.4 sessions (SD 17.3) over the duration of the treatment period. The mean session time per participant was 0.49 hours (SD 0.41), which amounts to a total mean exposure to the active treatment of 9.22 hours (SD 10.57). However, examining the standard deviation of this total time on the platform would suggest a large variance (ie, 10.57 hours). To illustrate this data further, quartiles have been reported in Table 3.

### Multiple Imputation

The Little MCAR test revealed a nonsignificant result (*χ*^2^_16_=10.8, *P*=.82), indicating that the data were missing completely at random and not contingent on any measured variables within the data set. Further exploration of the data indicated that no other variables (education level, gender, employment, BDI-II baseline score, GAD-7 baseline score, WSAS baseline score) were significantly different between participants who completed exit measures and participants who did not complete exit measures (*χ*^2^ and *t* tests). Similarly, these were not significantly different between participants who completed 3-month measures and participants who did not complete 3-month measures.

### Posttreatment and Follow-Up Effects

Linear mixed-model ANOVAs were conducted separately on the participant data (N=67) using time as the within-subjects variable for the BDI-II, GAD-7, and WSAS. For the BDI-II, a significant time effect on depression was found. The BDI-II scores significantly decreased from baseline with a mean of 35.94 (SD 6.91), to posttreatment with a mean of 23.76 (SD 9.68), to 3-month follow-up with a mean 14.52 (SD 7.45) measurement points, (*F*_2,132_=174.9, *P*<.001). Suicidality, as per question 9 on the BDI-II, was also found to significantly decrease across the measurement time points (*F*_2,132_=18.5, *P*<.001). Similar trends were observed in regards to the analyses of secondary measures, where significant decreases in scores were also observed for the GAD-7 (*F*_2,132_=98.3, *P*<.001), and significant increases for WSAS (*F*_2,132_=33.0, *P*<.001). Descriptive statistics are presented in Table 4.

### Reliable Change

Reliable change for the imputed data of the “severely” depressed group was explored using participants that had BDI-II data at both pre- and posttime points. At posttreatment, 43/67 (64%) participants were classified as reliably improved. Of those who were classified as reliably improved, 6 of the 67 (9%) participants met the criteria for recovery. The remaining 18/67 (36%) individuals were classified as unchanged, where scores did not exceed a movement of 9 points in either direction.

**Table 3:** Treatment response data (N=67).

Variable	Descriptive statistics			Percentiles		
	Mean (SD)	Median	Range (min-max)	25th	50th	75th
Number of sessions	17.4 (17.3)	13	85 (1-86)	5	13	25
Total time on platform (hours)	9.22 (10.57)	4.8	44.8 (0.0-44.8)	1.7	4.8	12.4
Time per session (hours)	0.49 (0.41)	0.4	2.9 (0.0-2.9)	0.3	0.4	0.6
Number of activities completed	33.4 (41.6)	16	227 (1-228)	5	16	51
Activities per session	1.79 (1.23)	1.5	5.7 (0.3-6.0)	0.9	1.5	2.1
Percentage of program viewed	0.46 (0.36)	0.4	1 (0.0-1.0)	0.2	0.4	0.8

**Table 4:** Descriptive statistics of the sample (n=67).

Measure	Baseline, mean (SD)	Posttreatment			3-month follow-up		
		Mean (SD)	95% CI (lower-upper)	Effect size *d* (95% CI)	Mean (SD)	95% CI (lower-upper)	Effect size *d* (95% CI)
BDI-II^a^	35.9 (6.9)	23.7 (9.7)	21.40-26.12	1.14 (0.77-1.51)	14.5 (7.4)	12.70-6.34	2.29 (1.85-2.73)
BDI-II Q9^b^	0.8 (0.6)	0.5 (0.6)	0.39-0.66	0.35 (0.01-0.69)	0.3 (0.5)	0.17-0.43	0.61 (0.26-0.96)
GAD-7^c^	13.6 (4.9)	8.0 (4.4)	6.91-9.09	0.90 (0.54-1.26)	6.00 (3.8)	5.05-6.92	1.32 (0.95-1.70)
WSAS^d^	20.4 (8.1)	15.2 (7.0)	13.49-16.93	0.53 (0.19-0.88)	12.8 (5.3)	11.49-14.09	0.84 (0.48-1.20)

^a^BDI-II: Beck Depression Inventory-II.

^b^BDI-II Question 9: Beck Depression Inventory Question 9 measuring suicidality.

^c^GAD-7: Generalized Anxiety Disorder-7 item inventory.

^d^WSAS: Work and Social Adjustment Scale.

## Discussion

### Principal Findings

The current paper sought to investigate the impact of an internet-delivered CBT intervention for the treatment of depression, *Space from Depression,* on symptoms of severe depression. The BDI-II was observed to have significant decreases with large effect sizes from pre- to post and 3-month follow-up timepoints. The results are encouraging, with participants demonstrating solid changes from pre-post assessment, and the changes beyond that time are suggestive of a continued positive change in symptom reduction. Baseline differences between the overall sample (N=211) and those who consented (n=67) found that those who consented had higher education levels, but did not vary across other clinical or sociodemographic variables. This difference in education is indicative of the wider literature; individuals with higher levels of education are more likely to participate in research [47], potentially stemming from increased levels of volunteerism [48] and belief in science [49] in this population. Treatment response rates for the study were also good, with 50/67 (75%) of participants completing 5 or more sessions.

The results demonstrate comparative effect sizes of improvement in severe depressive symptoms to those experienced in a mild to moderately depressed group, supporting the use of iCBT for severe depression [14,19]. Over half of the participants, 43/67 (64%) who provided pre- and posttreatment data experienced reliable improvement by participating in the intervention, supporting the potential for iCBT as an effective measure in this population. However, mean scores on the BDI-II remained within the moderately depressed range at posttreatment indicating that the majority of participants would still require further psychological treatment, despite improvement in their depressive symptomatology. These results are consistent with the conclusions of Bower et al [14], where although clinically reliable change was observed in the sample, the majority of those improved were still within clinical ranges (37/67, 55%) and would require further treatment. Further, despite no control group being recruited, limiting the statements that can be made in regards to effectiveness, this severe sample was found to have actively used the intervention over their course of treatment. This provides some support for the relationship between the intervention and the outcomes being reported.

Outcomes on secondary measures of the GAD-7 and WSAS significantly decreased from pre- to post timepoints, with significant differences and large effect sizes observed. These scores did not significantly differ at follow-up points, implying a maintenance of gains across the sample from post-treatment to 3-month follow-up, similar to what was observed by Meyer et al [19]. The decrease in anxiety scores as a result of iCBT treatment for depression was mirrored in the larger RCT this sample was derived from [28]. Reduction of comorbid symptoms in severe depression may be of particular importance to this population given the association that has been found between the severity of depressive symptoms and greater instances of comorbid conditions [50].

Increasingly in depression research, the need to include data points relevant to quality of life and functional impairment has been recognized [51,52]. The current study utilized the WSAS, and administering measures such as this can provide researchers and practitioners with deeper insight into current levels of experienced impairment and the recovery process. As a multi-domain scale encompassing areas such as work, leisure activities and home life, the improvements observed in the WSAS illustrate the positive consequences of a low-intensity iCBT intervention outside of decreasing depression symptoms. Other studies utilizing iCBT have recorded similar improvements in their samples [53-55], yet qualitative investigations may be necessary to understand the impact of iCBT on improvements in functioning further. Although preliminary, these results provide support for the helpfulness of iCBT, where an effective intervention should demonstrate its ability to produce outcomes not just on disorder symptomatology, but also in the areas of life typically affected by the disorder.

Clinically significant change is an important concept due to its potential to provide a complete picture of the impact of interventions, which goes beyond the averages of the group and outlines change at an individual level [56]. Conducting reliable change analyses also allows for researchers to be confident that changes in the score are more than fluctuations around a data point and, to an extent, regression to the mean. The approach adopted in this study was conservative and robust, and the reliable change analysis in research completers illustrated that 43/67 (64%) individuals reliably improved, with the remainder being unchanged. Six of the 67 (9%) individuals recovered using the criteria of <10 and a 9-point change on the BDI-II. The results further show potential for the use of internet-delivered interventions for severe populations, where a large proportion of individuals in a cohort with severe presentations achieved meaningful reductions in symptoms, and in some cases transitioning to mild-moderate categories from severe.

Suicidal ideation was significantly reduced from pre- to postintervention. The authors acknowledge that despite mean scores being less than 1 on this item of the BDI-II (where a score of 1 on the measure indicates the presence of suicidal thoughts without intentions), the results nonetheless lend support to previous trials investigating this area [57]. The results provide preliminary evidence for the feasibility of online interventions as effective means to tackle the increased risk of suicide in depressed populations.

The current study demonstrated the potential for a supported internet-delivered intervention to alleviate symptoms in individuals with severe presentations. Implementing supported iCBT interventions allows for sufficient monitoring of patients and the capability to intervene and offer further support in the case where risk escalates [19]. It is relatively unknown whether individuals with severe depression benefit more, or less from support than those in the mild to moderate range, though recent analyses have suggested that there is no difference in how populations of severity benefit from low-intensity interventions [14,58]. Further research would be helpful to delineate mechanisms associated with internet-delivered interventions and how they fluctuate depending on the population and severity.

### Limitations

While the initial results from this study are promising, the original protocol meant that no control group was recruited and therefore caution is advised as we are unable to conclude that the treatment delivered is responsible for the observed effects. We received a response rate of 37.1% (67/211) of the sample, which can be considered a limitation and represents a potential for self-selection bias. At baseline, we found no significant differences between responders and non-responders on all clinical and socio-demographic variables apart from education. Higher levels of education were found for participants who consented for their follow-up data to be used for this secondary analysis. The authors acknowledge that this may bias the data. However, it has been highlighted that differences in education do not impact on treatment response and outcome in CBT-therapies [59]. The result highlights the need for further investigation to discern the mediating and moderating effects of education and other variables on outcome from iCBT treatment for severe depression.

Further investigations implementing RCT or feasibility designs are warranted to discern the utility of *Space from Depression* in more severe cohorts, where our findings must also be understood within the context of the relatively small sample which gave consent for their data to be reported. Research regarding the long-term effects of an online intervention on severe depression would also be an important consideration. Participants’ depression and anxiety symptoms were indexed by self-report rather than clinical diagnosis. However, the authors employed standardized self-report measures that are well-established and have previously been used in numerous research studies relating to depression and internet-delivered interventions. Another potential limitation lies in the fact that the intervention was designed for a mild to moderately depressed group, and so, there may be some aspects that are less relevant for those with severe depression. A final limitation of the study is the large amount of missing data that was observed at posttreatment and 3-month follow-up for these individuals, and the authors, therefore, acknowledge that the results and their generalizability should be interpreted with caution.

### Conclusion

The current study demonstrated the potential for an internet-delivered intervention to reduce symptoms of severe depression. The participants demonstrated reliable decreases in anxiety symptoms and improvements in work and social functioning. Furthermore, reliable improvement in depression symptomology was observed. Suicidal ideation was reduced as a result of engaging in the intervention, and these results suggest that internet-delivered interventions may have the potential to provide a robust method of risk assessment and monitoring. Current treatment guidelines, such as NICE [13], recommend iCBT as a low-intensity treatment option for mild to moderate presentations of depression and advise high-intensity treatments for severe forms of depression. The present findings provide preliminary evidence to justify further research into the utilization of iCBT and the SilverCloud intervention as part of a treatment plan for severe depression that could prove beneficial for some individuals.

## Abbreviations

ANOVA: analysis of variance
BDI-II: Beck Depression Inventory
CBT: cognitive behavioral therapy
DSM-IV: The American Psychiatric Association Diagnostic and Statistical Manual of Mental Disorders (Fourth Edition)
GAD-7: Generalized Anxiety Disorder-7 item inventory
iCBT: internet-delivered cognitive behavioral therapy
NICE: The National Institute for Health and Care Excellence
RCT: randomized controlled trial
WSAS: Work and Social Adjustment Scale
